# The 150 most important questions in cancer research and clinical oncology series: questions 57–66: Edited by *Chinese Journal of Cancer*

**DOI:** 10.1186/s40880-017-0249-9

**Published:** 2017-10-03

**Authors:** Chinese Journal of Cancer

**Affiliations:** Sun Yat-sen University Cancer Center, Guangzhou, 510060 Guangdong P. R. China

**Keywords:** Antiangiogenic therapy, Stress, Lung cancer, MicroRNA, Renal cancer, Metastasis, Vasculogenic mimicry, EGFR, VEGFR, ERBB2, MET

## Abstract

Since the beginning of 2017, *Chinese Journal of Cancer* has published a series of important questions in cancer research and clinical oncology, which sparkle diverse thoughts, interesting communications, and potential collaborations among researchers all over the world. In this article, 10 more questions are presented as followed. Question 57. What are the major stresses that drive the formation, progression, and metastasis of a cancer? Question 58. What is the mechanism responsible for altering an acidic intracellular pH and a basic extracellular pH in normal tissue cells to a basic intracellular pH and an acidic extracellular pH in cancer cells, a fundamental and yet largely ignored phenomenon? Question 59. Where are the tumor-associated plasma microRNAs from in cancer patients? Question 60. Can we identify mechanisms employed by tumor subpopulations to evade standard therapies and seed relapse/metastatic tumors before treatment? Question 61. Why are mutation rates in epidermal growth factor receptor (*EGFR*) and erb-b2 receptor tyrosine kinase 2 (*ERBB2*) higher in lung cancer from never smokers than that from smokers? Question 62. Does tumor vasculogenic mimicry contribute to the resistance against antiangiogenic therapy in renal cancer? Question 63. What molecular targeted drugs would be effective for non-clear cell renal cell carcinoma (RCC), especially metastatic papillary RCC and chromophobe RCC? Question 64. Can it be more effective by targeting both the vascular endothelial growth factor receptor (VEGFR) and MET signaling pathways in sporadic metastatic papillary renal cell carcinoma (RCC)? Question 65. What are the predictive biomarkers that may be used to identify the renal cell carcinoma (RCC) patients who can benefit from immune checkpoint inhibitor treatment? Question 66. How do we identify predictive molecular biomarkers to stratify clear cell renal cell carcinoma patients for targeted therapies?

## Text

To accelerate our endeavors to overcome cancer, *Chinese Journal of Cancer* has launched a program of publishing 150 most important questions in cancer research and clinical oncology [1]. Since the beginning of 2017, *Chinese Journal of Cancer* has published a series of important questions in cancer research and clinical oncology [2–9], which sparkle diverse thoughts, interesting communications, and potential collaborations among researchers all over the world. In this article, Questions 57–66 are selected and presented. This program of collecting and publishing the key questions is still ongoing. Please send your thoughtful questions to Ms. Ji Ruan via email: ruanji@sysucc.org.cn.

## Question 57: What are the major stresses that drive the formation, progression, and metastasis of a cancer?

### Background and implication

One widely accepted theory about how gene mutations may drive cancer development is that certain gene mutations may give competitive edges of host cells over neighboring cells, leading to their proliferation. This argument may not hold for normal human tissue cells since cells do not compete in non-growing tissues [10]. Furthermore, there are various tissue-level constraints forbidding cells to proliferate in a well-organized tissue structure, such as contact inhibition and anchorage dependence. Hence, the tissue must be first in a condition under which competitions are allowed, e.g., damaged tissue under repair. Even under such conditions, there is no published research, to the best of our knowledge, that has demonstrated that proliferating cells indeed have a competitive edge over other cells, measured in well-defined metrics such as nutrient efficiency. Actually, published studies have demonstrated that cancerous cells tend to grow faster under more harsh conditions, including more hypoxic conditions [11, 12]. Therefore, it is reasonable to hypothesize that mutations in pre-cancerous cells are selected to better cope with certain stresses rather than simply having a competitive edge in a normal tissue condition. Published studies have identified that cancer and cancer-forming cells are under a variety of stresses, such as hypoxia, oxidative stress, and increasing intracellular pH [12–14]. However, it has been yet to establish which of these or other stresses or their combinations are the cancer-defining stresses that drive the underlying cells to select specific mutations for survival. Answering this question would improve our understanding on cancer biology and eventually improve cancer prevention and treatment.

#### Submitters

Sha Cao and Ying Xu.

#### Affiliation and emails

Computational Systems Biology Lab, Department of Biochemistry and Molecular Biology and Department of Statistics, University of Georgia, Athens, GA, USA.

shacao@uga.edu; xyn@uga.edu.

## Question 58: What is the mechanism responsible for altering an acidic intracellular pH and a basic extracellular pH in normal tissue cells to a basic intracellular pH and an acidic extracellular pH in cancer cells, a fundamental and yet largely ignored phenomenon?

### Background and implication

It has been well established that normal human cells have a slightly acidic intracellular pH and a mildly basic extracellular pH, but cancer tissue cells have the opposite [15]. The traditional explanation has been that certain proton exporters, such as NHEs and MCTs, are used to secrete protons or HCO_3_^−^ importers, e.g., NBCs, to drive up the intracellular pH for optimal performance by ribosome in support of efficient proliferation [16]. However, this argument may not hold since these transporters are driven by cross-membrane gradients of protons. Hence, the best that such transporters can accomplish is to have the intracellular and extracellular pH reach the same level. Furthermore, published studies have well established that these proton transporters, such as NHEs [17, 18] and MCTs [19], can move protons both inward and outward, depending on the direction of the gradients. In addition, the fact that cancer cells have these transporters highly up-regulated strongly suggests the possibility that these transporters move protons into cancer cells rather than secrete them as widely believed. An additional evidence is that V-ATPase, an ATP-powered proton importer that moves protons from extracellular space into cancer cells, is generally up-regulated in cancer, hence further indicating that the traditional argument is probably not correct. All these strongly suggest that there are some mechanisms in cancer cells that continuously produce basic elements to keep their intracellular pH being basic even under the condition that multiple transporters move protons into their intracellular space. Therefore, we raise a question: if proton exporters are not the main reason for the acidic extracellular pH and basic intracellular pH, what is the mechanism to make this fundamental change from normal cell to cancer cells? Answering this question would improve our understanding on cancer biology and hopefully shed some light on novel cancer treatment.

#### Submitters

Sha Cao and Ying Xu.

#### Affiliation and emails

Computational Systems Biology Lab, Department of Biochemistry and Molecular Biology and Department of Statistics, University of Georgia, Athens, GA, USA.

shacao@uga.edu, xyn@uga.edu.

## Question 59: Where are the tumor-associated plasma microRNAs from in cancer patients?

### Background and implication

Colorectal cancer (CRC) remains a major cancer type and contributes to cancer-related death worldwide [20]. Carcinoembryonic antigen (CEA) has been used as a serum marker of CRC. In our recently published study, we found that plasma microRNA-141 (miR-141) is a novel biomarker that complements CEA in detecting colon cancer with distant metastasis and that high levels of miR-141 in plasma are associated with poor prognosis [21]. These findings suggest that plasma-specific miRNAs have potential use as novel biomarkers of cancers and may be useful in clinical management for cancer patients.

However, the origin of these extracellular miRNAs remains to be elusive and yet to be fully elucidated. In the case of plasma miR-141 in metastatic CRC, we did not observe increased miR-141 level in metastatic CRC tumor tissues nor did we observe increased miR-141 level in white blood cells from these patients. Some studies showed that microRNAs (miRNAs) in plasma were released from cells in membrane-bound vesicles which are named microvesicles (exosomes) [22, 23]. These early reports are confirmed by observations that cultured cells release exosomes containing miRNAs [24]. Perhaps metastatic CRC cells secrete exosomes that are loaded with miR-141 into circulation quickly and thus enriched in the plasma.

Therefore, although it is a consensus that plasma miRNA markers are clinically valuable, it is necessary to systematically explore the origin of plasma miRNAs of cancer patients, which should give us a clue to the cancer biology and potential novel therapeutic strategy.

#### Submitters

Lina Zhang^1^, Kexin Chen^1^, and Wei Zhang^2^.

#### Affiliation and emails

^1^Tianjin Medical University Cancer Hospital and Institute, Tianjin, P. R. China.

^2^Wake Forest Baptist Comprehensive Cancer Center, Winston-Salem, NC, USA.

linazhang2005@126.com, chenkexin@tjmuch.com, wezhang@wakehealth.edu.

## Question 60: Can we identify mechanisms employed by tumor subpopulations to evade standard therapies and seed relapse/metastatic tumors before treatment?

### Background and implication

Cancer is a leading cause of death worldwide. A vast majority of cancer-related deaths are caused by relapse/metastatic diseases. Moreover, while we have significant improvement in treatments targeting primary tumors in many types of cancer, systematic treatment options for relapse or metastatic tumors are often less effective. Therefore, it is crucial to understand mechanisms and processes employed by relapse and metastatic tumors. Recent technical advances have made it possible to reveal intra-tumoral heterogeneity at a single-cell level. It is important for us to integrate single cell-based experimental platforms, proper disease models, and well-designed computational approaches to identify the tumor subpopulation(s) that evades the standard systematic therapy and eventually seeds relapse/metastatic tumors even before treatment. In addition, this approach will potentially reveal specific stromal components supporting the development of drug-resistant subpopulations. Overall, the knowledge should facilitate the development of innovative cancer therapies.

#### Submitter

Xiang Chen.

#### Affiliation and email

Departmental of Computational Biology, St. Jude Children’s Research Hospital, Memphis, TN, USA.

xiang.chen@stjude.org.

## Question 61: Why are mutation rates in epidermal growth factor receptor (*EGFR*) and erb-b2 receptor tyrosine kinase 2 (*ERBB2*) higher in lung cancer from never smokers than that from smokers?

### Background and implication

Lung cancer is a leading cause of cancer mortality worldwide and tobacco smoking is the most recognized causal factor for lung cancer. However, approximately 25% of lung cancers occur in lifelong never-smokers. With tobacco cessation campaigns successfully carried out in many countries, the proportion of never smokers with lung cancer has been increasing in recent years [25]. In Western countries, 10%–15% of all lung cancers in both men and women occur in never-smokers; non-small cell lung cancer (NSCLC) is particularly fascinating in women in Asian countries, and approximately 84% of Asian women are never smokers [26].

The mutation spectra of lung cancer in smokers and nonsmokers have been extensively studied and reported [27]. *EGFR* and Kirsten rat sarcoma viral oncogene homolog (*KRAS*) gene mutations and anaplastic lymphoma kinase (*ALK*) rearrangements are 3 major recurrent oncogenic alterations associated with lung cancer in never-smokers. Specifically, *EGFR* mutations have been reported in approximately 50% of never-smoker lung cancer patients compared with 10% of smoker lung cancer patients [28]. In non-small cell lung carcinoma, adenocarcinoma subtype, female never smokers were found to have a much higher frequency of *EGFR* gene mutations and echinoderm microtubule associated protein like 4 (*EML4*)-*ALK* transcript fusions than smokers. Therefore, these patients have significantly benefitted from specific targeted agents such as gefitinib and crizotinib [29]. *ERBB2* mutations, although occurring in a low frequency overall in lung cancer, also exhibited augmented mutation rate in non-smoker lung cancer patients [30]. These *ERBB2*-mutated lung cancer patients are good candidates for treatment with trastuzumab [31].

The emerging picture is that smoking-related cancers are characterized by a high mutation load due to tobacco-caused DNA mutagenesis [32]. These cancers have been recently shown to respond to anti-programmed death-1 (PD-1)/programmed death-ligand 1 (PD-L1) immunotherapy [33, 34]. The cancers in non-smokers tend to have a low mutation load and may not respond to immunotherapy. However, these tumors are shown to have increased *EGFR* and *ERBB2* mutations, thus may benefit from targeted therapies.

We therefore raise a question: Why are *EGFR* and *ERBB2* genes rarely mutated in lung cancers from smokers? A reasonable hypothesis for exploration in this direction is that a smoker’s lung might generate a physiological environment that favors tumors with a high mutation load while inhibiting *EGFR* and *ERBB2* signaling that is activated in non-smoking-related cancers. Answering this question would greatly improve our understanding on lung cancer biology and accelerate our efforts to identify more targetable molecules for lung cancer prevention and therapy.

#### Submitters

Meng Yang^1,2^ and Wei Zhang^1^.

#### Affiliation and emails

^1^Wake Forest Baptist Comprehensive Cancer Center, Winston-Salem, NC, USA.

^2^Tianjin Medical University Cancer Hospital and Institute, Tianjin, P. R. China.

wezhang@wakehealth.edu, meyang@wakehealth.edu.

## Question 62: Does tumor vasculogenic mimicry contribute to the resistance against antiangiogenic therapy in renal cancer?

### Background and implication

Antiangiogenic agents including sunitinib, sorafenib, temsirolimus, and pazopanib are currently used as the first-line therapy for advanced or metastatic clear cell renal cell carcinoma (ccRCC). Indeed, sunitinib has prolonged overall survival of ccRCC patients. Unfortunately, not all patients respond to antiangiogenic agents, and the vast majority of the patients eventually develop resistance to antiangiogenic therapy. A complete understanding of the mechanisms underlying cancer cell resistance against antiangiogenic agents are thus critical. One currently discussed mechanism is vessel co-option, by which the tumor-induced extra-tumoral angiogenesis followed by hijacking the newly formed extra-tumoral vessels becomes a portion of tumor vasculature [35].

Herein, we focus on another mechanism, namely, tumor vasculogenic mimicry. Tumor vasculogenic mimicry is the formation of vascular channels by tumor cells or tumor trans-differentiated cells in highly aggressive solid tumors including ccRCC. More importantly, in our exploration, we found that, under pharmacologically relevant concentrations, sunitinib could effectively inhibit the proliferation of normal endothelial cells but not ccRCC trans-differentiated endothelial cells. Thus, we hypothesize that vasculogenic mimicry may contribute to the resistance of ccRCC against antiangiogenic therapy.

The exploration on the resistant roles of vasculogenic mimicry against antiangiogenic treatment would broaden our knowledge and eventually improve the treatment efficacy on ccRCC.

#### Submitter

Jun-Ping Yang.

#### Affiliation and email

Sun Yat-sen University Cancer Center, State Key Laboratory of Oncology in South China, Collaborative Innovation Center of Cancer Medicine, Guangzhou, China.

yangjp@sysucc.org.cn.

## Question 63: What molecular targeted drugs would be effective for non-clear cell renal cell carcinoma (RCC), especially metastatic papillary RCC and chromophobe RCC?

### Background and implication

Thanks to the development of basic and translational researches on the major type of RCC, clear cell RCC, we have several approved targeted drugs available to extend patient survival, which are vascular endothelial growth factor receptor (VEGFR) inhibitors such as sunitinib, sorafenib, axitinib, and pazopanib and mammalian target of rapamycin (mTOR) inhibitors such as everolimus and temsirolimus. However, according to the reported guidelines, such as the European Association of Urology (EAU) and The National Comprehensive Cancer Network (NCCN) guidelines, there is no established targeted drug for non-clear cell RCC, especially metastatic papillary RCC and chromophobe RCC. The accumulating evidence shows that papillary RCC and chromophobe RCC have unique molecular characteristics distinct from those of clear cell RCC [36]. It is reasonable to hypothesize that novel specific targeted drugs could be developed for papillary and/or chromophobe RCC. Some promising explorations have shown that foretinib, which targets VEGFR and Met, may be effective for papillary RCC. Developing novel targeted drugs for papillary and/or chromophobe RCC would significantly prolong patient survival in addition to better understanding of the biology of RCC.

#### Submitter

Masayuki Takahashi.

#### Affiliation and email

Department of Urology, Institute of Biomedical Sciences, Tokushima University Graduate School, Tokushima, Japan.

takahashi.masayuki@tokushima-u.ac.jp.

## Question 64: Can it be more effective by targeting both the vascular endothelial growth factor receptor (VEGFR) and MET signaling pathways in sporadic metastatic papillary renal cell carcinoma (RCC)?

### Background and implication

There is no effective drug available for metastatic papillary RCC. Consequently, the patients with type 2 papillary RCC have a very poor prognosis. It has been found that hereditary papillary RCC harbors *MET* mutations. Sporadic papillary RCC does not frequently harbor *MET* mutations. However, recent comprehensive analyses of papillary RCC have shown that chromosome 7 gain and elevated expression of MET mRNA occur in sporadic type 1 papillary RCC. All of these findings suggest that targeting MET signaling in addition to targeting VEGFR signaling could be a promising strategy. Some promising evidence has been collected in a phase II clinical trial of foretinib, which targets multiple receptors in papillary RCC, including MET, vascular endothelial growth factor (VEGF), RON, AXL, and TIE-2 receptors. Cabozantinib, which is the same type of molecule-targeted agents as foretinib and has been approved for the treatment of advanced RCC in patients who have received prior anti-angiogenic therapy, may also be a promising agent for advanced papillary RCC. More endeavors are expected to answer this question for better tumor control [37].

#### Submitter

Masayuki Takahashi.

#### Affiliation and email

Department of Urology, Institute of Biomedical Sciences, Tokushima University Graduate School, Tokushima, Japan.

takahashi.masayuki@tokushima-u.ac.jp.

## Question 65: What are the predictive biomarkers that may be used to identify the renal cell carcinoma (RCC) patients who can benefit from immune checkpoint inhibitor treatment?

### Background and implication

In the recent clinical trial, about 20% of RCC patients can survive for a long time with the treatment with immune checkpoint inhibitor nivolumab, even after cessation of the treatment [38]. On the other hand, about 30% of patients have disease progression with nivolumab treatment [38]. Obviously, as immune checkpoint inhibitors are expensive and may cause severe immune-related adverse events in a certain number of patients, it should be administrated selectively to the right patients. However, there is no available biomarker that may be used to identify the right patients or to exclude the wrong patients for immune checkpoint inhibitors. More explorations should be pursued to identify the biomarkers for immune checkpoint inhibitors. We believe that significant survival benefits in RCC patients could be achieved by highly selective application of immune checkpoint inhibitors.

#### Submitter

Masayuki Takahashi.

#### Affiliation and email

Department of Urology, Institute of Biomedical Sciences, Tokushima University Graduate School, Tokushima, Japan.

takahashi.masayuki@tokushima-u.ac.jp.

## Question 66: How do we identify predictive molecular biomarkers to stratify clear cell renal cell carcinoma patients for targeted therapies?

### Background and implication

Kidney cancer, which is increasing in incidence, is associated with a high risk of death. About 80% of kidney cancers are clear cell renal cell carcinoma (ccRCC), which accounts for the vast majority of kidney cancer deaths. The 5-year overall survival rate for patients with metastatic RCC is only 20% [39]. Thus, improvement of current treatments of ccRCC, especially metastatic ccRCC, is urgently needed. Because ccRCC is notoriously resistant to chemotherapy and radiotherapy, the focus in recent years has been on targeted therapies as a critical option [39]. Ten targeted drugs have been approved by the Food and Drug Administration (FDA) of the United States for ccRCC, including six small-molecule inhibitors targeting angiogenesis and/or cell survival (sorafenib, sunitinib, pazopanib, axitinib, cabozantinib, and lenvatinib), two inhibitors of mammalian target of rapamycin (mTOR) (temsirolimus and everolimus), and two monoclonal antibodies targeting vascular endothelial growth factor (VEGF) (bevacizumab) or programmed death-1/programmed death-ligand 1 (PD-1/PD-L1) (nivolumab). Newer inhibitors of angiogenesis and other targets [such as platelet-derived growth factor receptor (PDGFR), fibroblast growth factor receptor (FGFR), c-kit, and MET] and monoclonal antibodies are being developed and tested in clinical trials for ccRCC [40]. More drugs targeting immune checkpoints are also being tested, alone or in combination with other drugs, in ccRCC. However, only a subset of patients benefit from current targeted therapies, and the benefit is usually short-term.

The utility of the ten approved targeted drugs and the numerous others in preclinical development for ccRCC is severely limited by the lack of validated predictive biomarkers that can be used to identify patients who are likely to benefit from a particular treatment. Researchers have identified subsets of ccRCC based on mRNA, microRNA, or protein profiles [41–43], but clinically relevant subtypes of ccRCC with associated prognostic and predictive biomarkers have not been identified and validated. No molecular biomarker has been used in the clinic. Such subtyping and biomarkers are of major importance and are urgently needed for patient selection in the clinic and for new drug testing in the preclinical setting.

With the rapid growth of information in omics studies on ccRCC and development of novel computational approaches, predictive molecular biomarkers of ccRCC will be identified by systemic analyses of the comprehensive information in the near future. Those biomarkers will be of significance to ccRCC patient care.

#### Submitter

Zhiyong Ding.

#### Affiliation and email

Department of Systems Biology, UT MD Anderson Cancer Center, Houston, Texas, USA.

zding@mdanderson.org.
